# The detrimental effects of smoking on the course and outcome in adults with bipolar disorder—A narrative review

**DOI:** 10.3389/fpsyt.2022.1114432

**Published:** 2023-01-09

**Authors:** Anna Grunze, Sergey Mosolov, Heinz Grunze, Christoph Born

**Affiliations:** ^1^Psychiatrisches Zentrum Nordbaden, Wiesloch, Germany; ^2^Moscow Research Institute of Psychiatry, Moscow, Russia; ^3^Russian Medical Academy of Continuous Professional Education, Moscow, Russia; ^4^Psychiatrie Schwäbisch Hall, Schwäbisch Hall, Germany; ^5^Paracelsus Medical University, Nuremberg, Germany

**Keywords:** bipolar disorder, depression, mania, nicotine, smoking, tobacco

## Abstract

**Background:**

Smoking is a substantial and avoidable risk for physical disability and premature death. Despite a declining tobacco use in the community of developed countries, smoking remains abundant in people with mental disorders. This narrative review highlights the epidemiology, consequences and treatment options of tobacco use disorder (TUD) and nicotine dependence (ND) in people with bipolar disorder (BD).

**Methods:**

The authors conducted a Medline literature search from 1970 to November 2022 using MeSH terms “bipolar disorder” x “smoking” or “nicotine” or “tobacco” that retrieved 770 results. Search results were complemented by additional literature retrieved from examining cross references and by hand search in text books. Finally, 92 references were considered as essential and selected for the educational purpose of this review.

**Summary of findings:**

Lifetime and point prevalence of smoking in people with BD is in the range of 45–70% and thus about 2–3 times more frequent in BD than in community samples. Smoking, TUD and ND have a detrimental impact both on mental and physical health as well as mortality in people with BD. In the absence of large controlled studies in comorbid BD and TUD or ND, pharmacological treatment follows the individual guidance for each disorder. Community-based psychosocial interventions for TUD and ND appear to be suitable in people with BD, too, as well as Cognitive Behavioral (CBT) or Acceptance and Commitment (ACT) based psychotherapies.

**Conclusions:**

Smoking is a modifiable risk factor causing increased risks both for mental and physical health in BD, and deserves more attention in treatment. More treatment research into pharmacological and psychosocial interventions in comorbid BD and TUD or ND are still needed to deliver evidence-based recommendations to physicians.

## 1. Introduction

Smoking is probably the leading avoidable cause of physical disability and premature mortality. Despite a declining tobacco use in the general population in developed countries, smoking remains abundant in people with mental disorders (1). But not only recreational use of tobacco is highly frequent, more worrisome is the fact that tobacco use disorder (TUD) and nicotine dependence (ND) is especially high in those suffering from severe mental disorders, including bipolar disorder (BD). A recent meta-analysis found that TUD or ND is prevalent in 33.4–65% of people with severe mental illness, with a male preponderance (2). Analyzing data from a population based US study, Lasser and colleagues reported adjusted odds ratios for both actual and life-time smoking in respondents with mental illness in the past month compared to healthy respondents of 2.7 (3). Smoking has a pronounced detrimental impact on well-being and life expectancy in people with severe mental illness including BD (4). A retrospective chart analysis from California found that tobacco-related health problems were responsible for ~48% of the death toll in people with BD (5). Furthermore, it also changes the course of BD and BD related disability such as functional impairment for the worse (6).

This review summarizes our current knowledge- and lack of- about epidemiology, physical and mental health impact and treatment of TUD and ND in people with BD.

## 2. Methods

This narrative review is based on a Medline search on November 12, 2022 using MeSH terms “bipolar disorder” x “smoking” or “nicotine” or “tobacco” retrieving 770 results. Results were further categorized by adding additional search terms: “etiology” or “etiology”, “treatment”, “randomized”, “mania” or “manic”, “depression” or “depressive”, “mixed”. Additional literature was retrieved examining cross references and by hand search in text books. Based on the authors' judgement, 92 articles were identified that contributed useful information for the educational purpose and have been included in this article. These papers were selected based on a consensus process of all authors after checking the total of 770 references and categorizing them by original research, meta-analyses, reviews and opinion papers. Duplication publications and reports that were considered either not to be of sufficient scientific rigor (e.g., single case reports) or containing information with little relevance to clinicians were excluded, the latter in order to keep the article of readable size.

## 3. Epidemiology of comorbid bipolar disorder and smoking

Synopsis of reported epidemiological data suggests that both lifetime and point prevalence of smoking in people with BD is in the range of 45–70% compared to 25–30% in the general population. Thus, compared to the general population, tobacco smoking is about 2–3 times more frequent in BD (1, 3, 7). However, smoking status in the general population varies significantly between countries, e.g., a small study suggests that more than twice as many adults smoke in Russia compared to the US (8). This means that a ceiling effect may be observed in some countries, blurring significant differences in numbers between smokers with mental health problems and the general population.

More in detail, data from the US National Comorbidity Survey indicate that the lifetime prevalence of daily smoking among adults with BD is 59%, and thus significantly higher in comparison to that of adults without mental disorders (39.1%) (3). A catchment area study from Kentucky reports that the prevalence of everyday current smoking is 66% in people with BD, which is also significantly elevated when compared to the general population, and also exceeds the rate observed in people with major depression and schizophrenia (9). In contrast, a systematic review of studies on smoking in severe mental disorders placed BD only second after schizophrenia but ahead of major depressive disorder (10). Using data derived from the general population as controls resulted in unadjusted odds ratios (ORs) for people with BD of 5.0 (95% CI: 3.3–7.8) for current cigarette smoking, 2.6 (95% CI: 1.7–4.4) for life-time cigarette smoking, and 0.13 (95% CI: 0.03–0.24) for stopping smoking, the latter indicating that the willingness to quit appears low in people with BD [albeit reports on psychosocial interventions suggest reasonable motivation (11)].

Finally, metanalytic analysis of three cross-sectional studies [one population based (12), two hospital based (13, 14)] also demonstrated a pooled point prevalence of TUD or ND among people with BD of 46.3 % (95 %CI = 33.9–59.2 %) (2).

Little data has been published differentiating between Bipolar I (BD-I) and Bipolar II (BD-II) patients. Results from the National Epidemiologic Survey on Alcohol and Related Conditions-III suggest a preponderance of DSM-5 nicotine use disorder (NUD) in people with BD-I. After controlling for sociodemographic characteristics and additional comorbid psychiatric disorders, Chou and colleagues (15) reported adjusted odds ratios for 12-month DSM-5 NUD of 1.93 (CI: 1.49–2.50) for BD-I, and 1.05 (CI: 0.67–1.64) for BD-II. For lifetime NUD, figures were 1.57 (CI: 1.29–1.90) for BD-I and 1.22 (CI: 0.77–1.95) for BD-II, respectively. These figures included any NUD, from mild to severe. Looking into severe NUD as defined by DSM-5 only, 12-month DSM-5 NUD adjusted odds ratio was 2.53 (CI:1.87–3.42) for BD-I, and 1.85 (CI:1.07–3.21) for BD-II.

## 4. Genetic predisposition

Besides environmental and epigenetic factors, there may be a genetic predisposition for smoking in people with BD and vice versa that explains, in part, the heightened incidence of TUD and ND in people with BD. There is a significant genetic overlap between people with addictive disorders and people with BD as demonstrated both by family studies (16) and shared polymorphisms of candidate genes involved in monoamine transport or metabolism (17, 18). Although ND is a predictor for developing BD (19), the genetic association, however, of ND and TUD with BD is less clear. In a recent genome-wide association study (GWAS) of smoking, smoking initiation and severity, and BD no genetic correlation could be established whereas smoking and schizophrenia appeared genetically correlated (20). However, with respect to BD, a single nucleotide polymorphism (SNPs) of the NR4A3 gene was found of special interest in another study (21) as it has a decisive role in the modulation of the dopaminergic responsiveness within the meso-cortical system. Association analysis of marker allele of the NR4A3 marker rs1131339 with smoking behavior in people with BD demonstrated a significant association with the risk of smoker status (smokers compared to non-smokers) as well as with the degree of smoking (21). This finding suggests that there is a genetic predisposition in a subgroup of people with BD for TUD.

Although the genetic association between TUD and ND and BD in general appears not unambiguous (22) there is an obvious interplay between smoking and psychotic BD. Analysis of the large database of the Genomic Psychiatry Cohort (GPC) revealed that among smokers, those with BD had a significantly increased risk of ND (OR = 2.5). People with BD with psychosis were on higher risk to be addicted than people with BD and no psychosis (OR = 1.3). People with BD experiencing higher severity of psychosis had also a higher risk of ND (23).

The increased risk of developing BD is not exclusive to active smokers. Of note, even maternal smoking while pregnant appears to raise the risk of BD two-fold in the off-springs (24).

## 5. Biological effects of smoking

**Figure 1:**
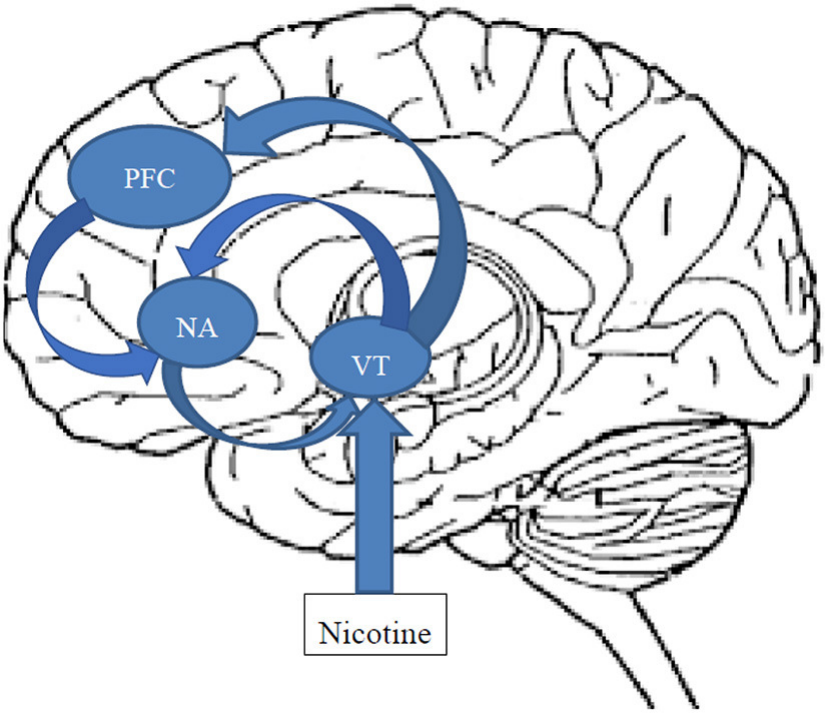
Schematic and simplified representation of the action of nicotine on the mesocorticolimbic dopaminergic reward circuitry. After passing the brain-blood barrier, Inhaled nicotine stimulates the low affinity α7 and the high affinity α4β2 nicotinic receptors located in the ventral tegmental area (VTA) activating the reward circuitry. Dopaminergic and glutamatergic neurons project from the VTA to the core and shell of the nucleus accumbens (NAC), as well as to the prefrontal cortex (PFC), glutamatergic neurons from the PFC to the NAC, and GABAergic neurons from the NAC back to the VTA. Both hyperexcitation of the VTA, e.g., by nicotine, as well as lack of inhibition by GABAergic projections from the NAC can result in rapid dopamine release.

Smoking enhances neuronal dopaminergic activity by at least two discrete modes of action. The central nicotinic cholinergic receptors, namely the low affinity α7 and the high affinity α4β2 nicotinic receptors located in the ventral tegmental area (VTA), are stimulated by nicotine resulting in a rapid release of dopamine in the nucleus accumbens, mediating the rewarding and reinforcing effects of nicotine (25) (Figure 1). In addition, stimulation of the VTA by nicotine has a regulating effect on serotonin release in the dorsal raphe mediated by inhibitory GABergic interneurons (26), and by this, also influences affect and emotion. Furthermore, cigarette smoke reduces Monoamine oxidase (MAO) A and B activity (27), prolonging the synaptic action of neurotransmitters with a role in mood regulation (e.g., serotonin and dopamine). In addition, nicotine has a cascading effect on central neurotransmission also resulting in the release of norepinephrine and histamine as well as GABA and glutamate (28, 29). Thus, it has been hypothesized that smoking is an effort to self-medicate in people with severe mental disorders (30), including BD, in terms of alleviating cognitive deficits (31), depression (32, 33) and reducing extrapyramidal symptoms associated with antipsychotic treatment.

These potential benefits of smoking, however, are contrasted by more severe detrimental effects. First, preliminary data from diffusion tensor imaging (DTI) studies report that smoking in young adulthood elevates fractional anisotropy (FA) among smokers compared to non-smokers (34), suggestive of impaired white matter development that might increase vulnerability to severe mental disorders. Once BD has manifested, the neuroprogression hypothesis in BD postulates that the recurrent mood swings might lead to growing damage in both neurons and glia, impairing the integrity of neuronal circuits and reinforce brain vulnerability to emerging mood episodes in the future (35), and finally leading to neurodegeneration (36). Nicotine and the free radicals contained in cigarette smoke activate several neurobiological effector systems implicated in the neuroprogression of BD (22). As far as dopamine is concerned, elevated brain dopamine concentrations also exert oxidative stress, due to the oxidative processes involved in dopamine metabolism: MAO produces H_2_O_2_ and dihydroxyphenylacetic acid through a catalytic dopamine reaction (37). In addition, tobacco smoke promotes neuroprogression in BD *via* several possible pathways leading to cumulative effects; particularly oxidative and nitrosative stress pathways where it increases the oxidative burden, further triggers reactive nitrogen species (RNS, e.g., nitric oxide) and reactive oxygen species (ROS) effects of excitotoxicity and mitochondrial dysfunction. Similar to acute BD episodes, tobacco smoke also activates inflammatory pathways, including the release of cytokine and enhancing cyclooxygenase 2 levels. This mechanism also makes smoking the most relevant biological mediator of co-morbid cardiovascular disease in people with BD (38). Besides monoaminergic, glutamatergic as well as neurotrophic pathways are also altered by both smoking and BD, pointing toward potential interactive effects (22). As an overall result, smoking is thought to accelerate neuroprogression in BD leading to the clinical consequences detailed below.

## 6. Smoking as a risk factor for bipolar disorder

Concerning the question whether TUD predisposes to BD, no prospective controlled study examined relative odds in smokers compared to non-smokers in developing BD. However, a bidirectional Mendelian randomization study tested *post-hoc* the impact of both smoking initiation and total lifetime smoking on risk of BD in a large sample of subjects participating in genome-wide association studies (GWAS, including 20,129 cases and 21,524 controls), using summary level data (39). Analyzing the results evidence emerged that smoking was an underlying risk factor for BD (OR = 1.46 for smoking initiation, 95% CI: 1.28–1.66, *p* < 0.001, and OR = 1.72 for lifetime smoking, 95% CI: 1.29–2.28, *p* < 0.001). Vice versa, there was no unambiguous evidence that having BD causally influenced the risk of smoking-related outcome parameters (39).

## 7. Consequences of smoking in people with bipolar disorder

### 7.1. Consequences for mental health

The impact of smoking on the course of BD becomes noticeable quite early. *Post-hoc* analyses of the “Course and Outcome of Bipolar Youth (COBY)” study revealed that smoking is a worrisome concern in adolescents with BD. Twenty-five percent of the study participants had ever smoked, with 11 % daily, and half of them had been heavy smokers (≥10 cigarettes per day). Compared to those adolescents with BD who never smoked, smoking status was associated with a greater prevalence of BD-I, earlier onset of BD, higher lifetime prevalence of physical and sexual abuse as well as suicide attempts, conduct disorder, and other substance use disorders (SUD). In addition, previous psychiatric hospitalizations were significantly more frequent among daily smokers vs. those who never smoked (40). Smoking as a risk factor for an earlier age of onset of BD was also found in an Italian study in more than 1,000 patients of specialized outpatient clinics (41) and in a clinical sample of 195 adolescents from the US with BD (42). Heavy smoking in adolescents with BD also paves the way to heavier marijuana use, illicit drug use disorders, and poorer overall functioning (13). In adults with BD, smoking has also been associated with higher symptom load for mania, depression and anxiety (43, 44), more agitation and impulsivity (45, 46), more hospitalizations, lower functionality and more suicide attempts (6, 47, 48). In addition, dimensional analysis in smokers with BD (number of cigarettes per day vs. different clinical variables) reports a positive correlation for number of daily cigarettes and age, age at onset of BD, illness duration, and a current diagnosis of comorbid panic disorder (44), the latter being a frequent comorbidity in BD.

Of note, not only smoking, but also cessation of smoking puts people with BD on increased mental health relapse risk. When stopping smoking, people with BD may also have a transient increased risk of a manic or depressive recurrence (49–51).

The relationship between BD and smoking might be bidirectional. A recent Finnish register study revealed that the mean count of cigarettes/day at age 16 were associated with a higher risk of subsequent manifestation of BD after adjustment for potential confounders (OR, 1.23 [95% CI 1.01–1.50]) (52). During times of ongoing neuronal maturation, adolescents may be especially sensitive to harmful effects of substance use. Two longitudinal studies indicate that alcohol use and cigarette smoking during adolescence may procure noxious neurobiological effects (33, 34).

### 7.2. Consequences for physical health

Worldwide, smoking is a leading cause of avoidable deaths in general (53), and it contributes considerably to premature mortality in BD. It has been estimated that almost half of deaths in people who have ever been hospitalized for BD result from tobacco-related health conditions (5). Across studies, people with BD have, on average, a life expectancy that is 15–20 years shorter than in the general population (54). In a large Swedish registry study, women and men with BD died 9.0 and 8.5 years earlier on average than the rest of the population, respectively (55). Keeping in mind that the smoking prevalence in Sweden is relatively low (only 11% of the Swedish population were daily smokers in 2016) these mortality figures are probably less affected by smoking and probably illustrate the gain in preventing premature mortality in BD that can be achieved with smoking cessation. Despite the increased proportion of unnatural deaths as suicide and accidental death, medical conditions account for about 70% of deaths in people with BD, with cardiovascular disease contributing 22.0% to the reduction in overall life expectancy in people with BD (54). Thus, cardiovascular illness may be the greatest driver of excess mortality in BD (56, 57), perhaps more so for females (58). As bipolar patients are also more frequently affected by other cardiovascular risk factors related to life style (little physical activity and poor diet, resulting in overweight and metabolic issues, the detrimental impact of smoking on cardiovascular health might be even more pronounced compared to people with a healthier life style (59, 60). Women with BD also have a higher mortality from cancer, and again smoking might be a contributing factor (55). Comparing life expectancy in people with BD and comorbid SUD, TUD has a similar negative impact as comorbid alcohol or opioid use disorder (4).

## 8. Treatment of comorbid bipolar disorder and tobacco use disorder

Data on willingness to quit smoking in people with severe mental illness are inconclusive; both less motivation (61) and similar motivation (62) compared to smokers without mental health problems have been reported.

Combined pharmacological and psychosocial treatment appears to be the most promising approach to quit smoking. A review of 24 studies of tobacco smoking cessation in people with mental disorder (schizophrenia, major depressive disorder, and SUD other than TUD), with most interventions consisting of both pharmacotherapy and psychoeducation, reported that smoking cessation rates for people with mental disorders were of similar magnitude than those expected for the general population (63). Unfortunately, similar data specifically for people with BD are scarce.

As with other SUD comorbid with BD, an integrated approach targeting simultaneously BD and TUD appears preferrable; however, evidence supporting this approach is still scarce. The fast majority of smoking cessation studies in people with severe mental disorders have been conducted in stable patients. For many individuals with BD established smoking cessation approaches may be sufficient. For others it may be more profitable to have smoking cessation treatments delivered by their familiar mental health care professional, using a personalized approach additional to the support provided by mainstream services (6).

### 8.1. Pharmacological treatment of bipolar disorder in people with tobacco use disorder or nicotine dependence

The pharmacological treatment of BD does not differ between smokers and non-smokers but some pharmacokinetic peculiarities need to be observed. Tobacco fume does not interfere with the most commonly used mood stabilizers lithium, valproate and lamotrigine. However, smoking might affect the metabolism of some other frequently used medication by inducing enzymes of the cytochrome P 450 family (CYP). Polycyclic aromatic hydrocarbons contained in Cigarette smoke increase the activity of CYP 1A2 enzymes, and thus affects the metabolism of its substrates, several antipsychotics, e.g., olanzapine, haloperidol, clozapine, as well as some antidepressants (amitriptyline, clomipramine, doxepin, duloxetine, fluvoxamine, imipramine, mianserin, mirtazapine) and benzodiazepines (6, 64). Dose adaption or even a change of medication might be indicated in smokers with BD, i.e., for olanzapine and clozapine a 50% higher dosage might be required (65). Treatment might be complicated by the fact that dopamine antagonists such as antipsychotics decrease pleasure, reinforcement and reward of tobacco smoking, which, in turn, might result in heavier smoking (66).

### 8.2. Pharmacological treatment of tobacco use disorder or nicotine dependence in people with bipolar disorder

There is an obvious paucity of profound research and, as a consequence, a relative lack of published studies on the psychopharmacological treatment of BD comorbid with TUD or ND. As a matter of fact, our systematic literature search revealed that not a single double-blind placebo-controlled study has been conducted exclusively in this population. In general, nicotine metabolism is similar across smokers with BD and healthy subjects. Nicotine is metabolized in the liver by the cytochromes (CYP) 2A6 (~0.90%) and CYP 2B6 (~10%) (67). People with BD taking hepatic enzyme-inducing drugs such as carbamazepine, oxcarbazepine or topiramate have an increased nicotine metabolism, possibly resulting in the urge of heavier smoking and making it more difficulty to quit smoking (68). This metabolic pathway also opens the door to interactions with some psychotropic medication metabolized by CYP 2A6, namely valproate, or CYP 2B6 (bupropion). Due to the large metabolic capacities of these cytochromes, interactions are only in exceptional cases of clinical relevance. As far as interaction with psychotropic drugs in concerned, polycyclic aromatic hydrocarbons within tobacco smoke play a more decisive role by inducing CYP1A2 (Table 1). The clinical significance of such interactions has been demonstrated for olanzapine and haloperidol treatment in a *post-hoc* analysis of the Phase 3 olanzapine mania studies by Berk and colleagues (69). Smokers and non-smokers with mania of comparable severity had diverging treatment outcomes, with those who continued smoking showing overall poorer clinical outcomes vs. those who did not smoke during the trials.

**Table 1:** Polycyclic aromatic hydrocarbons within tobacco smoke induce CYP 1A2 and accelerate metabolism of the listed psychotropic substrates.

**Antidepressants**	**Anxiolytics**	**Antipsychotics**	**Mood stabilizer**
**SSRI**	**BZD**	**FGA**	**AC**
Fluvoxamine	Alprazolam	Chlorpromazine	Valproate
**SNRI**	Chlordiazepoxide	Fluphenanzine	
Duloxetine	Diazepam	Haloperidol	
**DSA and NaSSA**	Lorazepam	Pimozide	
Trazodone	Oxazepam	Thiothixene	
Mianserin	Triazolam	**SGA**	
Mirtazepine		Clozapine	
**TCA**		Olanzapine	
Amitriptyline			
Clomipramine			
Doxepin			
Imipramine			
Nortriptyline			
**Others**			
Agomelatine			

Extent of interaction and, by this, clinical relevance varies as some substrates are very sensitive to CYP 1A, e.g., duloxetine, others only moderately, e.g., clozapine, or weakly, e.g., valproate. Additionally, many substrates are subject to two or more cytochrome isoforms for metabolism, or/and are unmetabolized excreted by the kidneys.

SSRI, selective serotonin re-uptake inhibitor; SNRI, Serotonin-norepinephrine re-uptake inhibitor; DSA, Dual serotonergic acting antidepressants (SSRI/5HT2 antagonist; NaSSA, Noradrenergic and Specific Serotonergic Antidepressant; TCA, tricyclic antidepressant; BZD, benzodiazepines; FGA, First generation antipsychotics; SGA, Second generation antipsychotics; AC: Anticonvulsants.

We identified only four published smoking cessation treatment trials in smokers with BD: one very small open pilot study with bupropion (70), two open pilot studies with varenicline (71, 72), and 2 studies using varenicline either alone (73) or in combination with CBT (74). All studies were in favor of the specific treatment. However, as this evidence base is quite limited, in clinical practice people with BD usually receive the same treatments for TUD or ND as smokers not affected by mental health disorders.

As there is some evidence that smoking might serve as self- medication against depressed mood (75), several antidepressants had been tested for smoking cessation. A Cochrane review and meta-analysis found high-certainty evidence that bupropion can support smoking cessation in the long-term. However, compared to placebo, bupropion also showed an increased rate of adverse events (AE), including psychiatric AEs. With lower evidence, nortriptyline, too, appeared to have beneficial effects on smoking cessation rates vs. placebo. For all other antidepressants, evidence for aiding smoking cessation was found to be insufficient or non-existing (76).

The partial nicotine agonist varenicline has been licensed in several countries for smoking cessation, based on several randomized controlled studies against placebo, bupropion and nicotine replacement patches (77). Of note, *in vivo* varencline also has antidepressant-like effects in the forced swim test, and is able to augment sertraline's antidepressant effect *via* its action on the α4β2 receptor. These beneficial effects may additionally be mediated *via* the α7 nicotinic receptor as this receptor subtype also enhances the antidepressant effects of serotonin uptake blockers (75).

Finally, nicotine replacement therapy (NRT, with gum, transdermal patch, intranasal spray and inhaled and oral preparations) has high-quality evidence that all of the licensed forms of NRT support people who want to quit smoking. NRT increases the rate of quitting by 50–60%, regardless of setting and NRT preparation (78).

A *post-hoc* analysis suggests a higher abstinence rate with varenicline compared to bupropion or NRT (79), but with all three pharmacological interventions (bupropion, varenicline, NRT) being, by large, equally effective for TUD or ND (80), safety comes to the fore. NRT has little contraindications or side effects, and can be considered in almost all people with TUD. Skin irritation my occur in nicotine patch users. Bupropion SR, however, should be used with caution in patients with liver or kidney disease, and other treatments should be preferred in patients with known seizures or who are at risk for seizures (81). Bupropion might occasionally induce treatment emergent affective switches, albeit with lower probability than several other antidepressants (82).The only contraindication for varenicline is allergy to the medication. Apart from this, nausea is the most frequent adverse effect. Also of importance is the fact that varenicline appears safe in patients with cardiovascular disease. Case reports on varenicline-induced mania exist mainly for elderly BD patients (83), but, in general, varenicline can be prescribed in patients with stable mental disorders (81).

### 8.3. Psychosocial smoking cessation interventions

As with other SUD comorbid with BD, an integrated approach targeting simultaneously BD and TUD appears preferrable; however, evidence supporting this approach is still scarce. No eligible meta-analyses of RCTs have been reported in a recent meta-review concerning the effectiveness of psychosocial smoking interventions for psychiatric symptoms in people with mental disorders (84). Conversely, some studies on psychosocial interventions in people with BD and TUD with the primary outcome “smoking cessation” do exist.

Heffner et al. explored a CBT-based mood management treatment in a small open study in people with BD and comorbid ND (85). The program consisted of 12 weekly sessions, 60 min each, using a manualized CBT intervention incorporating standard counseling (e.g., TUD relapse prevention methods, monitoring triggers for cigarette smoking) and mood control (e.g., cognitive re-structuring, coping with stress, maintaining regular sleep-wake schedules). After 4 weeks, all participants also received nicotine patches for the remaining 8 weeks of the study. At study endpoint, 20% of subjects managed to refrain from smocking for 4 weeks, and 77% of those completing the study achieved a reduction in self-reported cigarettes per day of 50% or higher. Of note, depression and mania scores showed small increment from baseline to post-treatment, possibly caused by nicotine withdrawal. But given the small number of subjects (*n* = 10) this may be just a random finding. Evaluating the feedback of participants, its appeared that 60 min sessions were perceived as too long, and the counseling part was not considered suitable for all participants because there was no flexibility to address other, quite heterogeneous reasons for smoking.

Incorporating these lessons learned from the pilot study, the same group (86) next tested ten sessions of a 30-min in-person (*n* = 10) and telephone-based (*n* = 6) Acceptance and Commitment Therapy (ACT) protocol for smokers with comorbid mild BD-I or BD-II. The focus is ACT's primary elements of acceptance and commitment (e.g., behavior driven by personal values). Sessions included mindfulness techniques (e.g., awareness of situations with cigarette craving and emerging mood symptoms in a non-judgmental manner), cognitive defusion (e.g., distancing self from troubling thoughts), and identification of personal principles and values being worth smoking cessation (e.g., love of next of kin). Treatment content and intensity was identical for both the in-person and telephone-based protocols. Again, nicotine patches to support abstinence were offered to participants after the third session. After the specific intervention, four-week abstinence rates were 30% for the in-person treatment and 17% for the telephone protocol group. Furthermore, in both protocols, at least 50% of participants managed to reduce number of cigarettes smoked per day by ≥50%. Different from the previous pilot trial, reported depressive and manic symptoms did not ameliorate.

Some studies tested examined the effects of community-based smoking cessation programmes on smokers with severe mental illness, but none specifically for people with BD (87, 88). A variety of psychosocial interventions were examined in these studies, including motivational enhancements, smoking cessation education, cognitive behavioral strategies, and contingency management. Meta-analysis of these studies showed that individually tailored face-to face interventions are superior to standard care for smoking cessation, but comparing bespoke digital on-line interventions vs. standard digital on-line interventions did not demonstrate any difference in effectiveness (88).

Successful smoking cessation treatment for people with severe mental disorders does not necessarily rely on expert centers but can also be delivered in community-based mental health services. A large Australian study reported a smoking cessation rate in this population of 15.3 %, comparable to what has been achieved in smokers without mental disorder. This open program consisted of recurring 2 h—sessions that supplied information according to participants' needs, including managing mental health, how to deal with boredom, sadness and stress, and acquirement of confidence and coping strategies. Likelihood to quit was higher for those who attended more sessions, had already decided at entry that they want to quit, and in those participants with a lower extent of ND. Cessation rates did not differ between males and females, nor were the number of years of smoking or the psychiatric diagnosis significantly correlated with the cessation rate (11).

## 9. Limitations

This article has been written for educational purposes and does not claim to be a full and comprehensive review of evidence, but summarizes key studies and reviews according to the author's judgement. It covers publication with a time span of more than 20 years. Not only the definition of BD (89), but also of what has historically been considered as “tobacco addiction” has been refined over the last decades (e.g., smoking habit, nicotine consumption, nicotine dependence, nicotine addiction), with varying meanings inconsistently used in published studies. The diagnosis of TUD as introduced by the Diagnostic and Statistical Manual for Mental Disorders, Fifth Edition (DSM-5) (90) now allows for a broader inclusion than past definitions. In addition, most studies looking at the prevalence of comorbid TUD or ND among people suffering from a severe mental disorder have not deployed validated structured interviews for establishing a diagnosis (91, 92). As a consequence, prevalence, correlates, and odds of comorbid TUD or ND among people with severe mental disorders, including BD, show a relatively wide range (2).

## 10. Conclusion

TUD and ND are about 2–3 times more frequent in persons with BD in comparison to community samples. Reasons may include a genetic predisposition, environmental factors and the stimulating effects of nicotine on the mesolimbic and mesocortical reward system. Smoking may constitute an attempt to self-medicate in terms of alleviating cognitive deficits, depression and reducing extrapyramidal symptoms associated with antipsychotic treatment. However, the detrimental effects of smoking on neuroprogression of BD and physical health outweigh these pleasurable effects by far. The pharmacological treatment of BD does not differ fundamentally between people with or without TUD but pharmacokinetic interactions between cigarette smoke and some medication need to be kept in mind. Limited evidence favors prescription of bupropion, varenicline or NRT for smoking cessation also in people with BD. Similar to pharmacological treatments, non-pharmacological interventions for smoking cessation in people with BD obviously require further research (2). Supplementing pharmacological treatment, ACT based psychotherapy appears the technique of choice, but also CBT based interventions and community-based smoking cessation programs, modified for use in people with severe mental disorders, yield some promise.

## Author contributions

AG completed the first draft of the manuscript which was amended and revised by CB, SM, and HG. All authors read and approved the final manuscript and consented for publication.
